# Reducing dose to the lungs through loosing target dose homogeneity requirement for radiotherapy of non small cell lung cancer

**DOI:** 10.1002/acm2.12200

**Published:** 2017-10-11

**Authors:** Junjie Miao, Hui Yan, Yuan Tian, Pan Ma, Zhiqiang Liu, Minghui Li, Wenting Ren, Jiayun Chen, Ye Zhang, Jianrong Dai

**Affiliations:** ^1^ Department of Radiation Oncology National Cancer Center/Cancer Hospital Chinese Academy of Medical Sciences and Peking Union Medical College Beijing China

**Keywords:** IMRT, non small cell lung cancer, plan optimization, target dose homogeneity, treatment planning

## Abstract

It is important to minimize lung dose during intensity‐modulated radiation therapy (IMRT) of nonsmall cell lung cancer (NSCLC). In this study, an approach was proposed to reduce lung dose by relaxing the constraint of target dose homogeneity during treatment planning of IMRT. Ten NSCLC patients with lung tumor on the right side were selected. The total dose for planning target volume (PTV) was 60 Gy (2 Gy/fraction). For each patient, two IMRT plans with six beams were created in Pinnacle treatment planning system. The dose homogeneity of target was controlled by constraints on the maximum and uniform doses of target volume. One IMRT plan was made with homogeneous target dose (the resulting target dose was within 95%–107% of the prescribed dose), while another IMRT plan was made with inhomogeneous target dose (the resulting target dose was more than 95% of the prescribed dose). During plan optimization, the dose of cord and heart in two types of IMRT plans were kept nearly the same. The doses of lungs, PTV and organs at risk (OARs) between two types of IMRT plans were compared and analyzed quantitatively. For all patients, the lung dose was decreased in the IMRT plans with inhomogeneous target dose. On average, the mean dose, V5, V20, and V30 of lung were reduced by 1.4 Gy, 4.8%, 3.7%, and 1.7%, respectively, and the dose to normal tissue was also reduced. These reductions in DVH values were all statistically significant (*P* < 0.05). There were no significant differences between the two IMRT plans on V25, V30, V40, V50 and mean dose for heart. The maximum doses of cords in two type IMRT plans were nearly the same. IMRT plans with inhomogeneous target dose could protect lungs better and may be considered as a choice for treating NSCLC.

## 1 INTRODUCTION

Lung cancer is the most frequently diagnosed cancer and the leading cause of cancer death among males. About 85% of lung cancers are NSCLC worldwide.^1^ Radiotherapy plays an important role in the treatment of locally advanced, unresectable NSCLC.^2^ Treatment outcome of standard radiotherapy for NSCLC patients has not improved much during the past decades, and the 5‐year relative survival rate is still no more than 20%.^3^ Local failure often occurs in the primary tumor. Doses higher than the standard 60–66 Gy are required to obtain a better local tumor control.^4^, ^5^ But this is limited by the protection of organs at risk. Some patients suffered from severe side effects after radiation therapy.^6^, ^7^ Radiation pneumonitis (RP) is the main dose‐limiting complication in radiation therapy for NSCLC, and occurs in 5%–50% of patients.^8^, ^9^ In the case of conventionally fractionated radiotherapy, the traditional strategy for minimizing patients' risk is to follow empirically established dose–volume constraints, such as V20 < 30%–35% and mean lung dose (MLD) <20–23 Gy. However, the relationship between RP risk and dosimetical statistics such as MLD varies among institutions. And it also changes when different treatment techniques (i.e., CRT, IMRT, and VMAT) are applied.^10^

IMRT is a common technology for the treatment of lung cancer.^11^ The target volume could get higher dose and better conformity index than 3D‐CRT. However, the improvement of uniformity of target dose could increase the volume of the low dose area in nearby OARs. As a result the volume of low dose area in the lung could be significantly increased.^12^, ^13^ Shirvani et al found that the proportion of patients under IMRT treatment increased year by year and V20 of lung decreased significantly in IMRT group based on 3986 patients. Compared with 3D–CRT group the adverse reaction of lung occurred at similar rates using IMRT and showed that the lower V20 did not reduce the incidence of RP.^14^ The reason may be that V5 of lung would be increased as IMRT applied.^15^ To minimize the risk of RP some new techniques were introduced to reduce the lung dose, including respiratory gated PET/CT, Cyber Knife, VMAT, etc.^16^, ^17^, ^18^, ^19^

Conventionally, the standard practice is that tumors should be irradiated to an intended uniform, or homogeneous, dose.^20^ While this optimizes the tumor control probability in the case of homogenous tumors, this is generally not the optimal dose distribution in tumors with spatial variation in radiation sensitivity.^21^ In addition, dose escalation strategies that involve delivery of uniform doses are typically limited by normal tissue dose tolerance. There have been studies indicating that deliberately using nonuniform radiation doses allows for dose escalation of tumor subvolumes without necessarily increasing the dose which is delivered to adjacent critical structures.^22^, ^23^

It still remains unclear whether the dose to OARs could be reduced when the dose nonuniformity in the target area is increased. The goal of this study is to investigate whether it is beneficial to decrease the lung toxicity for NSCLC by increasing target dose inhomogeneity in IMRT plans.

## 2 METHODS

### 2.A Patient data

10 NSCLC patients with lung tumor on the right side treated at our institution between July 2014 and October 2015 were selected in this study which had been approved by ethics committee. The patient characteristics are listed in Table 1. The patient‐related privacy information (e.g., name, identification number, telephone number) has been removed. All the patients were immobilized in the supine position using a thermoplastic mask. Treatment plans were made based on computed tomography (Philips, Brilliance Big Bore) with slice thickness of 3 mm.

**Table 1:** Summary of patient characteristics

Patient No.	Age	TNM	PTV Volume (cm^3^)
1	35	T2N3M0	392.9
2	73	T3N2M0	403.1
3	47	T2N1M0	210.8
4	60	T2N2M0	227.5
5	77	T3N1M0	273.4
6	55	T2N3M0	381.0
7	40	T2N1M0	212.9
8	69	T2N1M0	225.3
9	57	T2N2M1	251.6
10	56	T3N2M0	395.8

The gross tumor volume (GTV) was delineated by experienced radiation oncologists based on the planning CT (free‐breathing scan) with fluorodeoxyglucose positron emission tomography (FDG‐PET) images as auxiliary references, and consisted of the primary tumor and involved lymph nodes. The clinical target volume (CTV) was created as an expansion of the GTV by 10 mm in mediastinum and 5 mm in lung tissue excluding bony structures and major vessels in accordance with recommendations provided by the Danish Oncology Lung cancer Group (DOLG). The planning target volume was created as a patient specific expansion of the CTV with a margin of 5–10 mm.^24^, ^25^ The organs at risk included the heart, the spinal cord, the lungs, and the normal tissues. The prescribed dose was 60 Gy in 2.0 Gy daily fractions.

### 2.B Plan optimization

The IMRT plans were made on Pinnacle 9.10 workstation and treated on Elekta Synergy accelerator using 6 MV photon beams. The adaptive convolution algorithm provided by Pinnacle was chosen as the dose calculation engine and the calculation grid resolution was set as 2 × 2 × 2 mm^3^. The dose of treatment plans was calculated on free‐breathing CT. Two IMRT plans using a static step‐and‐shoot delivery approach were created for each patient. One was with standard homogeneous dose distribution (IMRT_homo_) for target, and the other was with an inhomogeneous dose distribution (IMRT_Inho_) for target. The two IMRT plans used the same couch and collimator angles, and consisted of six beams with the gantry angles 185°, 215°, 245°, 345°, 15°, and 155° respectively.

For IMRT_homo_ plans, PTV dose was restricted within 95%–107% of the prescribed dose (60 Gy) based on the recommendation of International Commission on Radiation Units. 26 The maximum dose of spinal Cord PRV (planning organ at risk volume) was 45 Gy. V_20 Gy_ and mean dose of the lungs were set to be as low as possible. The homogeneity of the PTV was enforced by increasing the weight of the PTV uniform dose to 100. For IMRT_inho_ plans, the dose constraints were: PTV dose ≥95% of the prescribed dose (60 Gy), maximum dose of 45 Gy to the spinal Cord PRV, V_20 Gy_ and mean dose for the lungs were set to be as low as possible. There were no uniform dose constrains for PTV. For each patient, a “Boost” optimization region was constructed by expanding the GTV with the same expansion margin. There were no limitations on the maximum dose to this region. Details of objectives set for the initial optimization were illustrated in Table 2.

**Table 2:** Objective settings for the initial optimization

Item	ROI name	Group	Objective type	Target(Gy)	Weight
1	PTV	IMRT_homo_	Maximum dose	63	50
	PTV‐Boost	IMRT_inho_	Maximum dose	63	50
2	PTV	IMRT_homo_	Uniform dose	60.5	100
		IMRT_inho_	/	/	/
3	PTV	Both	Minimum dose	59.5	90
4	PTV	Both	Minimum DVH	60/95%coverage	100
5	PTV‐3 mm	Both	Minimum dose	60	30
6	Lung	Both	Maximum DVH	5/44% coverage	30
7	Lung	Both	Maximum DVH	20/18% coverage	60
8	Lung	Both	Maximum DVH	30/14% coverage	30
9	Lung	Both	Maximum EUD	11	1
10	Cord	Both	Maximum dose	35	40
11	Cord PRV	Both	Maximum dose	38	60
12	Cord PRV	Both	Maximum EUD	7	0.5
13	Heart	Both	Maximum DVH	30/18% coverage	30
14	Heart	Both	Maximum DVH	40/13% coverage	30
15	Heart	Both	Maximum EUD	15	0.3
16	Ring1	Both	Maximum dose	59	20
17	Ring2	Both	Maximum dose	56	20
18	Normal Tissue	Both	Maximum dose	50	20
19	Normal Tissue	Both	Maximum EUD	10	0.2

Minimum DVH 60/95% coverage: the minimum normalized volume that is radiated by a dose greater than 60 Gy is 95%; Maximum DVH 5/44% coverage: the maximum normalized volume that is radiated by a dose greater than 5 Gy is 44%.

The additional contours were as follows: (a) PTV‐3 mm, shrinkage from the PTV by 3 mm; (b) Ring1 and Ring2, the 5‐mm‐wide rings at 5 mm and 10 mm distance, respectively, from the PTV; (c) Normal Tissue, the whole CT volumes excluding the PTV expanded by 20 mm in all directions; (d) Boost, GTV expanded by 2 mm in all directions; (e) PTV‐Boost, the volume of PTV excluding Boost.

### 2.C Plan evaluation

IMRT_homo_ and IMRT_Inho_ plans were considered acceptable if at least 95% of PTV volume receiving 100% of the prescribed dose. All plans were reviewed and evaluated by one experienced radiation oncologist according to the standard clinical protocol. To quantify the target coverage and dose distribution, various dosimetric metrics were applied as follows: (a) D_2%_ defined as the maximum dose for the PTV and indicated the maximum dose, and D_98%_ indicated the minimum dose; (b) conformity index (CI) and homogeneity index (HI); (c) total number of monitor units; (d) mean dose. For a fair comparison, we normalized IMRT_inho_ and IMRT_homo_ plans to the same level of D_98%_ of PTV. Organs at risk were evaluated in terms of the D_mean_ and percent of volumes receiving different doses. The percent of volumes V5, V10, V15, V20, V30, V40, V50, and D_mean_ for Lungs and V25, V30, V40, V50, and D_mean_ for heart were recorded. The maximum dose and D_mean_ for cord, cord PRV and normal tissue were also recorded. A margin of 5 mm was added to cord to form the cord PRV.

Normal tissue complication probabilities (NTCP) model was used to evaluate the treatment plans. The model was based on the Lyman–Kutcher–Burman(LKB) model in this study. LKB model is defined with the following equations: 27

(1) $$\mathrm{NTCP}=\frac{1}{\sqrt{2\pi }}\int_{-\infty }^{t}e^{-\frac{x^{2}}{2}}dx$$

(2) $$\frac{t=D_{eff}-TD_{50}}{mTD_{50}}$$

(3) $$D_{eff}={\left(\sum_{i}v_{i}D_{i}^{1/n}\right)}^{n}$$

where *D*_*eff*_ is the dose that, if given uniformly to the entire volume, will lead to the same NTCP as the actual nonuniform dose distribution, *TD*_50_ is the uniform dose given to the entire organ volume that results in 50% complication risk, *m* is the slope of the curve represented by the integral of the normal distribution, *n* is a parameter which describes the magnitude of the volume effect, *v*_*i*_ is the relative volume related to dose voxel *D*_i_. In this calculation, *TD*_50_ = 24.5 Gy, *m* = 0.18, and *n* = 0.87 for lung which is given by Pinnacle planning system. All the results were analyzed using the paired *t*‐test. A *P*‐value of less than 0.05 was considered statistically significant. SPSS software (IBM SPSS Statistics, version 22) was used for the analyses.

## 3 RESULTS

**Figure 1:**
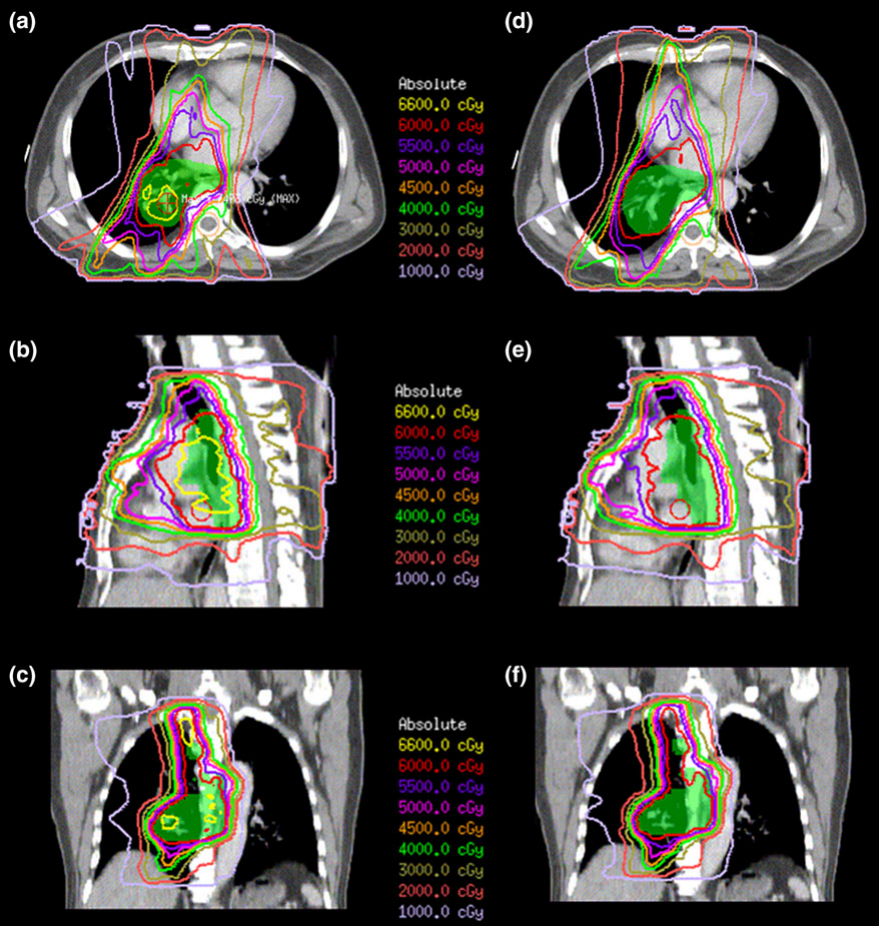
Isodose distribution of IMRT_inho_ (a, b, c) and IMRT_homo_ (d, e, f) plans for one patient with six coplanar beams.

**Figure 2:**
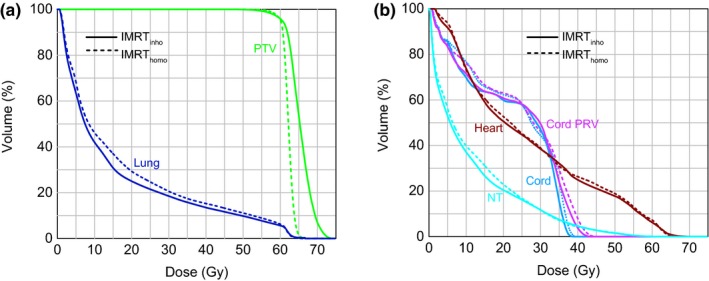
Dose–volume histograms (DVH) for (a) PTV and Lung (b) Heart, Cord, Cord PRV, and NT obtained with IMRT_inho_ and IMRT_homo_ plans.

For all patients, both IMRT_homo_ and IMRT_inho_ plans were accepted for clinical treatment by the radiation oncologist. Figures 1 and 2 were the comparisons of dose distributions and dose–volume histograms between two plans for one patient. They showed that the uniformities of PTV dose of two plans were different. It was also clear that the mean dose of lung in IMRT_inho_ plan was lower than that in IMRT_homo_ plan, while the doses of the other OARs in IMRT_inho_ plans were nearly the same as those in IMRT_homo_ plans.

The dosimetric statistics of PTV in the two IMRT plans were listed in Table 3. IMRT_homo_ exhibited better HI than IMRT_inho_ (*P* < 0.001). D_2%,_ Mu and mean dose showed significant difference with *P* < 0.001, *P* = 0.001, and *P* < 0.001 respectively. There were no significant differences for D_98%_(*P* = 0.876), CI (*P* = 0.176), number of segments (*P* = 0.364), and delivery time (*P* = 0.094) when comparing IMRT_homo_ plans with IMRT_inho_ plans.

**Table 3:** Dosimetric parameters comparison of homogeneous and inhomogeneous plans for PTV (mean ± standard deviation)

PTV	IMRT_homo_	IMRT_inho_	*P*‐value
D_2%_ (Gy)	64.7 ± 1.6	70.1 ± 2.3	<0.001
D_98%_ (Gy)	58.3 ± 0.7	58.3 ± 0.7	0.876
CI	0.66 ± 0.04	0.70 ± 0.06	0.176
HI	0.11 ± 0.04	0.19 ± 0.04	<0.001
MU	419.7 ± 76.1	498.0 ± 61.3	0.001
Mean (Gy)	62.3 ± 0.7	66.0 ± 1.9	<0.001
Segments	37.2 ± 8.4	38.7 ± 6.9	0.364
Delivery time(s)	323 ± 26	352 ± 32	0.094

CI, conformity index; $CI=\frac{TV_{RI}\times TV_{RI}}{TV\times V_{RI}}$ where TV_RI_ is the target volume covered by the 95% prescription dose, TV is the target volume and V_RI_ is the volume of the 95% prescription dose. HI, homogeneity index; HI = (*D*_2_–*D*_98_)/*D*_prescription_; MU, monitor units.

**Figure 3:**
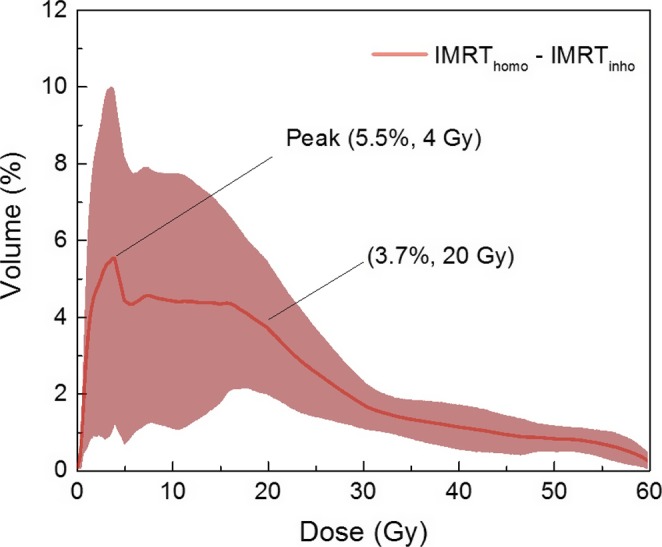
Averaged difference of dose–volume histograms (IMRT_homo_–IMRT_inho_) for lung with standard deviation. The standard deviation has been drawn as lines with fill area.

The dosimetric statistics of OARs in the two IMRT plans were listed in Table 4. The mean volume of lungs in this study was 3013.3 cm^3^ (ranged from 2571.4 to 4892.3 cm^3^). Lung dose in IMRT_inho_ plans was significant reduced. V20 of lungs decreased from 27.9 ± 3.8% (IMRT_homo_) to 24.2 ± 3.1% (IMRT_inho_) with *P* < 0.001, and the MLD decreased from 15.7 ± 2.1 Gy (IMRT_homo_) to 14.3 ± 2.0 Gy (IMRT_inho_) (*P* < 0.001). On average, V5 of lungs decreased from 57.1 ± 8.3% (IMRT_homo_) to 52.3 ± 8.0% (IMRT_inho_). V10 of lungs decreased from 42.6 ± 7.1% (IMRT_homo_) to 38.2 ± 6.8% (IMRT_inho_), and V30 of lungs decreased from 19.6 ± 2.4% (IMRT_inho_) to 17.9 ± 2.4% (IMRT_inho_). Other dose–volume statistics such as V15, V40, and V50 in IMRT_inho_ plans also showed significant decreases while comparing with those in IMRT_homo_ plans. Figure 3 showed averaged difference of dose–volume histograms (IMRT_homo_ – IMRT_inho_) for lungs with standard deviation. The peak of averaged difference was 5.5% corresponding to the dose of 4 Gy in the low dose area.

**Table 4:** Dosimetric parameters comparison between homogeneous and inhomogeneous plans for organs at risk (mean ± standard deviation)

Organ at risk	IMRT_homo_	IMRT_inho_	*P*‐value
Lung			
V5 (%)	57.1 ± 8.3	52.3 ± 8.0	0.005
V10 (%)	42.6 ± 7.1	38.2 ± 6.8	0.002
V15 (%)	34.4 ± 5.7	29.9 ± 4.5	<0.001
V20 (%)	27.9 ± 3.8	24.2 ± 3.1	<0.001
V30(%)	19.6 ± 2.4	17.9 ± 2.4	<0.001
V40 (%)	14.2 ± 2.4	13.1 ± 2.7	0.001
V50 (%)	9.5 ± 2.7	8.7 ± 2.9	0.001
Mean (Gy)	15.7 ± 2.1	14.3 ± 2.0	<0.001
NTCP	4.2 ± 1.5%	2.5 ± 1.2%	<0.001
Heart			
V25(%)	25.1 ± 10.6	24.5 ± 12.3	0.543
V30(%)	21.6 ± 9.3	21.3 ± 10.8	0.687
V40(%)	14.4 ± 6.4	14.9 ± 8.4	0.662
V50(%)	8.7 ± 4.2	9.1 ± 5.7	0.733
Mean (Gy)	16.1 ± 5.8	16.0 ± 7.0	0.869
Cord			
Max (Gy)	37.7 ± 5.0	37.6 ± 5.8	0.860
Cord PRV			
Max (Gy)	42.1 ± 4.1	42.9 ± 4.7	0.273
Normal tissue			
Mean (Gy)	12.0 ± 1.2	11.2 ± 1.3	0.002

For heart, the differences of V25, V30, V40, and V50 values between IMRT_homo_ and IMRT_inho_ plans were within 0.6%, and all the *P* values of statistical parameters were more than 0.05. Therefore, there were no significant differences between the IMRT_homo_ and IMRT_inho_ plans on V25, V30, V40, V50 and mean dose. As to cord and cord PRV, the maximum dose were 37.7 ± 5.0 Gy and 42.1 ± 4.1 Gy in IMRT_homo_ plans, while those were 37.6 ± 5.8 Gy and 42.9 ± 4.7 Gy for IMRT_inho_ plans. And the *P* values were 0.860 and 0.273, indicating the maximum dose of cord and cord PRV in the both plans had no significant difference. For normal tissue, IMRT_inho_ plans showed lower doses than IMRT_homo_ plans. The mean doses in the two plans were 12.0 ± 1.2 Gy and 11.2 ± 1.3 Gy with *P* = 0.002.

## 4 DISCUSSION

Nielsen et al have used inhomogeneous dose distributions as a way to increase tumor control probability.^28^ The goal of this study was to investigate if increasing target dose inhomogeneity could be beneficial to decrease the lung toxicity for NSCLC using IMRT plans. For this purpose, IMRT_inho_ plans were developed with no uniform dose constraints for PTV and no maximum dose constrains for GTV. As shown in Table 3, CIs of the two IMRT plans were nearly the same and HIs were significantly different. For IMRT_homo_ plans mean dose to the PTV was 62.3 ± 0.7 Gy, whereas for IMRT_inho_ plans that was 66.0 ± 1.9 Gy. In certain cases, IMRT_inho_ plans could increase the dose to tumor. Although the increased dose was only about 3.7 Gy, it might be beneficial to improve local control of tumor.

The correlation between severe RP and MLD was reported by several authors.^29^, ^30^, ^31^ In a planning study by Murshed, it was suggested that if MLD was decreased by 2 Gy the risk of RP could be reduced by 10%.^32^ It was reported that introducing V5 constraint could significantly decreased the lethal pneumonitis. Claude et al showed that only MLD, V20 and V30 were predictive of the severe RP.^33^ They suggested that a large panel of thresholds from low to high dose could provide advantages.

The result shown in Fig. 3 and Table 4 suggested that lower lung dose values and NTCP could be obtained from low to high dose region in the IMRT_inho_ plans. Relaxing target dose homogeneity could lead to a significant reduction (3.7% absolute difference) in the lung volume receiving doses <20 Gy which was in the low dose region. IMRT_inho_ plans showed an average decrease of V5 with 4.8% absolute difference compared with IMRT_homo_ plans. Therefore, decreased volume of lung was irradiated at low dose area. In the high dose region, IMRT_inho_ plans could give a dose reduction to lung by about 10%. So the risk of radiation pneumonitis could potentially be decreased. The mean dose, V30 and V40 were often used as indicators for heart toxicity. The equal heart toxicity was expected for both homogeneous and inhomogeneous plans in this study because their mean dose, V30 and V40 were similar. The maximum dose of cord and cord PRV in the two plans were kept similar. While lung dose reduced, normal tissues dose was also decreased in IMRT_inho_ plans. This study demonstrated that reducing dose to lungs was possible without increasing the dose to the other OARs.

**Figure 4:**
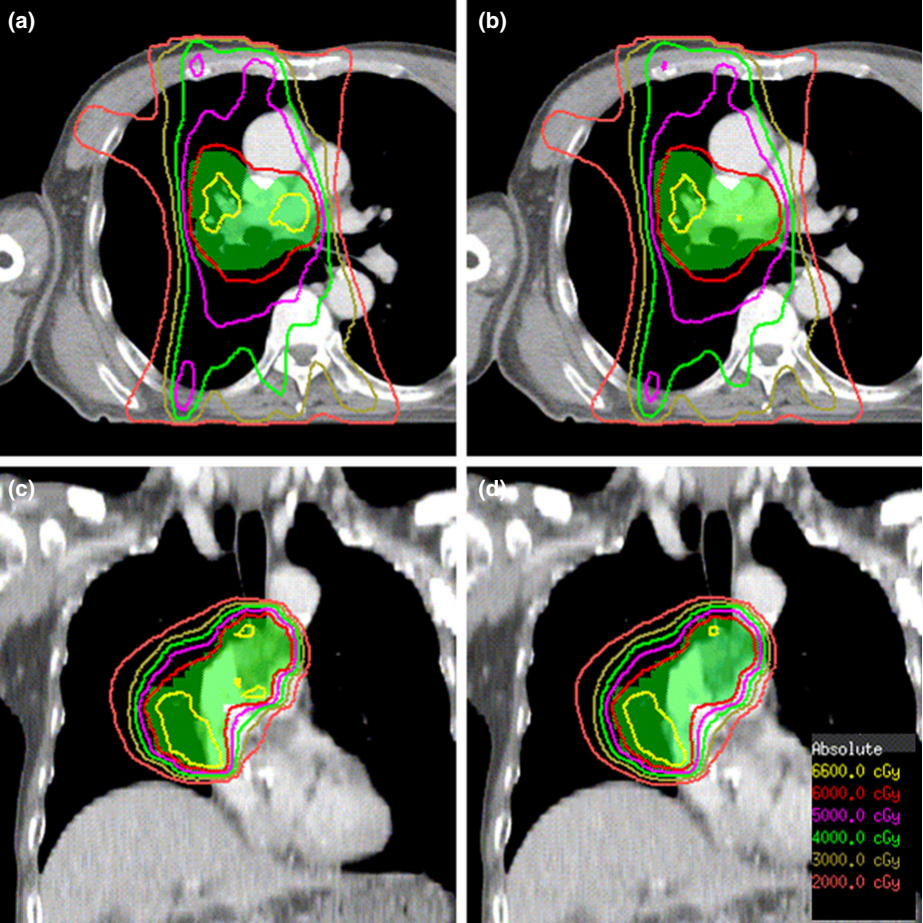
Dose distribution of IMRT_inho_ and IMRT_inho_+5 mm plans.

**Figure 5:**
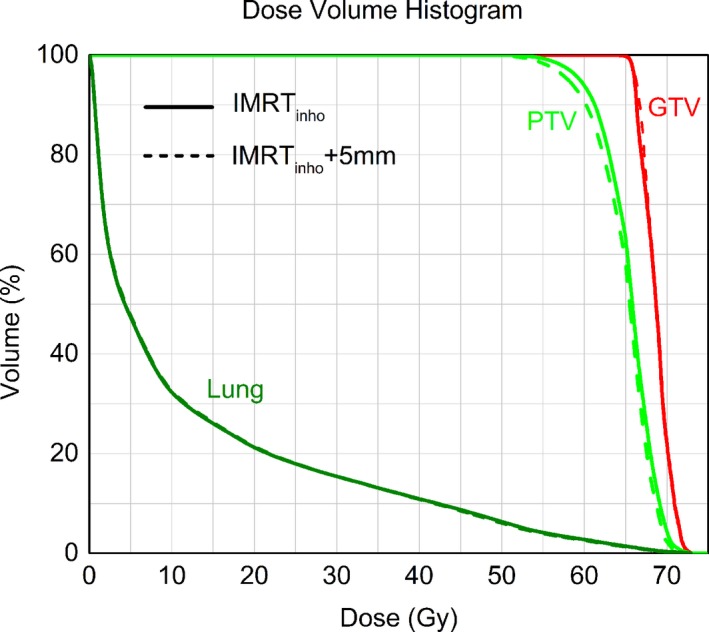
Dose–volume histograms for PTV, GTV, and Lung obtained with IMRT_inho_ and IMRT_inho_+5 mm plans.

The esophagus was often located close to or may be part of the target volume. In this case, plan optimization might lead to high dose at the esophagus which must be avoided by adding more constraints to restrict it. The hot spots which were close to cord should be paid special attention and these hot spots should be avoided as possible in the process of plan optimization. The impact of daily setup errors on dose distribution was estimated by modifying the position of the isocenter point on planning CT slices of one patient. Six plans with same beamlets and MUs of IMRT_inho_ were created, and the prescribed dose was 10 Gy in 2.0 Gy daily fractions for each plan. Isocenter was shifted toward the head, foot, left, right, anterior, and posterior, respectively, by 5 mm in each plan. The total “estimated” plan (IMRT_inho_+5 mm) was composed of the six plans. Dose distribution between IMRT_inho_+5 mm and IMRT_inho_ plans was compared as shown in Fig. 4. We can see that the position of hot spots remains unchanged and big hot spots become smaller due to edge blurring effect from the Fig. 4. Some small hot spots that appear in IMRT_inho_ plan don't manifest in the dose distribution received by the patient due to day to day setup variations. There is little change in lung and GTV doses between IMRT_inho_ plans and IMRT_inho_+5 mm plans as shown in Fig. 5.

Respiratory motion and tumor shrinkage throughout treatment can further affect the dose to the tumor and the hot spots may move toward nearby critical structures. The use of daily localization with CBCT could detect changes in the target and ensure the safety of OARs.^34^ As the spatial distribution of biological properties in tumors such as proliferation and hypoxia are known to be nonuniform, the dose distribution that maximizes tumor control probability for a given tumor is also nonuniform. Regions of high radiosensitivity may correspond to regions of high tumor proliferation, whereas regions of high radioresistance may correspond to regions of tumor hypoxia. Based on positron emission tomography images and IMRT_inho_ plans, these regions might be controlled to get inhomogeneous dose distribution to increase tumor control probability. Besides NSCLC, this method should be valid in other cancers such as esophageal cancer, liver cancer, etc., which will be investigated in the future studies.

## 5 CONCLUSION

It was demonstrated that relaxing the constraints on maximum and uniform dose in the target volume could significantly reduce the dose to lungs from low to high dose region. With this approach, the risk of radiation pneumonitis could potentially be decreased. In addition, it could increase the mean dose to tumor, which might be beneficial to improve local control of tumor by radiotherapy. IMRT plans with inhomogeneous target dose could protect lungs better and may be considered as a choice for NSCLC treatment.

## CONFLICT OF INTEREST

All authors approved the final manuscript, and declared that they have no potential conflicts of interest to this work.
